# I-European research, the cradle of the discovery of the antidiabetic hormone: the pioneer roles and the relevance of Oskar Minkowski and Eugène Gley

**DOI:** 10.1007/s00592-022-01976-y

**Published:** 2022-10-14

**Authors:** Alberto de Leiva-Hidalgo, Alejandra de Leiva-Pérez

**Affiliations:** 1 Instituto Interuniversitario López Piñero, University of Valencia, Valencia, Spain; 2 Universidad Autónoma de Barcelona, Barcelona, Spain; 3 Fundación DIABEM, Barcelona, Spain

**Keywords:** Organotherapy, Diabetes, Pancreatectomy, Pancreatic extracts, Antidiabetic hormone

## Abstract

**Aims:**

The introduction of hormonal treatment in severe diabetes in 1922 represented a clinical and social impact similar to that of antibiotic therapy. In October 1923, the Assembly of the Karolinska Institute decided to award the Nobel Prize in Physiology or Medicine to the Canadian Frederick Grant Banting and the Scottish John James Rickard Macleod, researchers at the University of Toronto (UT), for "the discovery of insulin a year before". A few weeks later, European and American researchers protested the decision. The controversy remains to this day.

**Methods:**

We have conducted a comprehensive review of primary and critical sources focused on the organotherapy of animal and human diabetes mellitus since 1889, when Oskar Minkowski demonstrated the induction of experimental diabetes by total pancreatectomy in the dog, until the spring of 1923, when the Nobel Foundation had already received all the nominations for the award in Physiology or Medicine.

**Results:**

The in-depth analysis of all these sources revealed that Europe was the cradle of the discovery of the antidiabetic hormone. The discovery involved multiple research steps headed by a long list of key investigators, mainly European.

**Conclusion:**

Marcel Eugène Émile Gley was the first to demonstrate the presence of the “antidiabetic principle” in extracts from “sclerosed” pancreas. The French physiologist pioneered the successful reduction of glycosuria and diabetic symptoms by the parenteral administration of pancreatic extracts to depancreatized dogs in experiments developed between 1890 and 1905, antedating insulin in two decades.

## Pancreas: exocrine gland

**Fig. 1:**
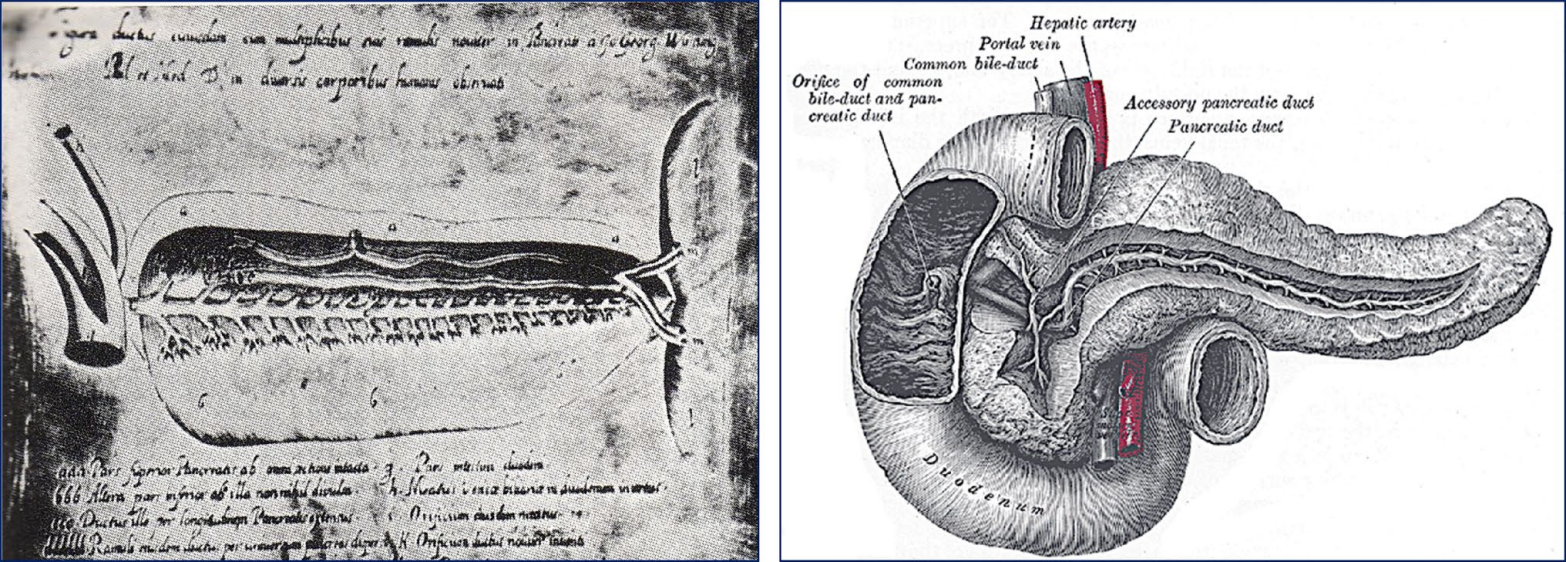
*Left image*: F.G. Wirsüng (1642). Original copperplate exhibited in the Palazzo del Bò, Padua (Edizione Universitarie Patavine, Padua) [1]. *Right image*: Illustration of the pancreatic-duodenal area. Henry Gray (1918) Anatomy of the Human Body, Fig. 1100. Provo, Utah. Brigham Young University: Lea & Fibiger; p. 1202. Internet Archives. https://www.bartleby.com/107/illus1100.html

The discovery in 1642 of the main pancreatic duct by Johann Georg Wirsüng (1589–1643) in Padua was decisive for the subsequent definition of the pancreas as an important secretory gland in the process of digestion. Wirsüng, who was unaware of the functions of the gland and its main duct, never published the discovery. He engraved an anatomical drawing of the pancreas on copper plates. The original plate is exhibited in the Palazzo del Bo in Padua. In 1654 Francis Glisson described the sphincter muscle surrounding Vater's ampulla; in 1711, the anatomy of the ampulla was detailed by Abraham Vater (1684–1761). In 1887, Ruggero Oddi (1864–1913) characterised the circular and longitudinal muscle fibres wrapped around the end of the bile and pancreatic ducts, which justifies it being known as the sphincter of Oddi. The first description of the accessory duct has been attributed to Giovanni Domenico Santorini (1681–1737), not without some controversy. Franciscus Sylvius (Frans de le Boë), professor at the University of Leiden, and teacher of De Graaf, also published the existence of the second duct (Fig. 1) [1, 2].

Claude Bernard's scientific work between 1843 and 1878 comprised more than three hundred scientific papers. Most of them were reports of his laboratory experiments presented to scientific societies, mainly the Académie des Sciences and the Société de Biologie. Other important contributions were his lectures given to students at the Collège de France, the Sorbonne and the Musée d' Histoire Naturelle [3].

**Fig. 2:**
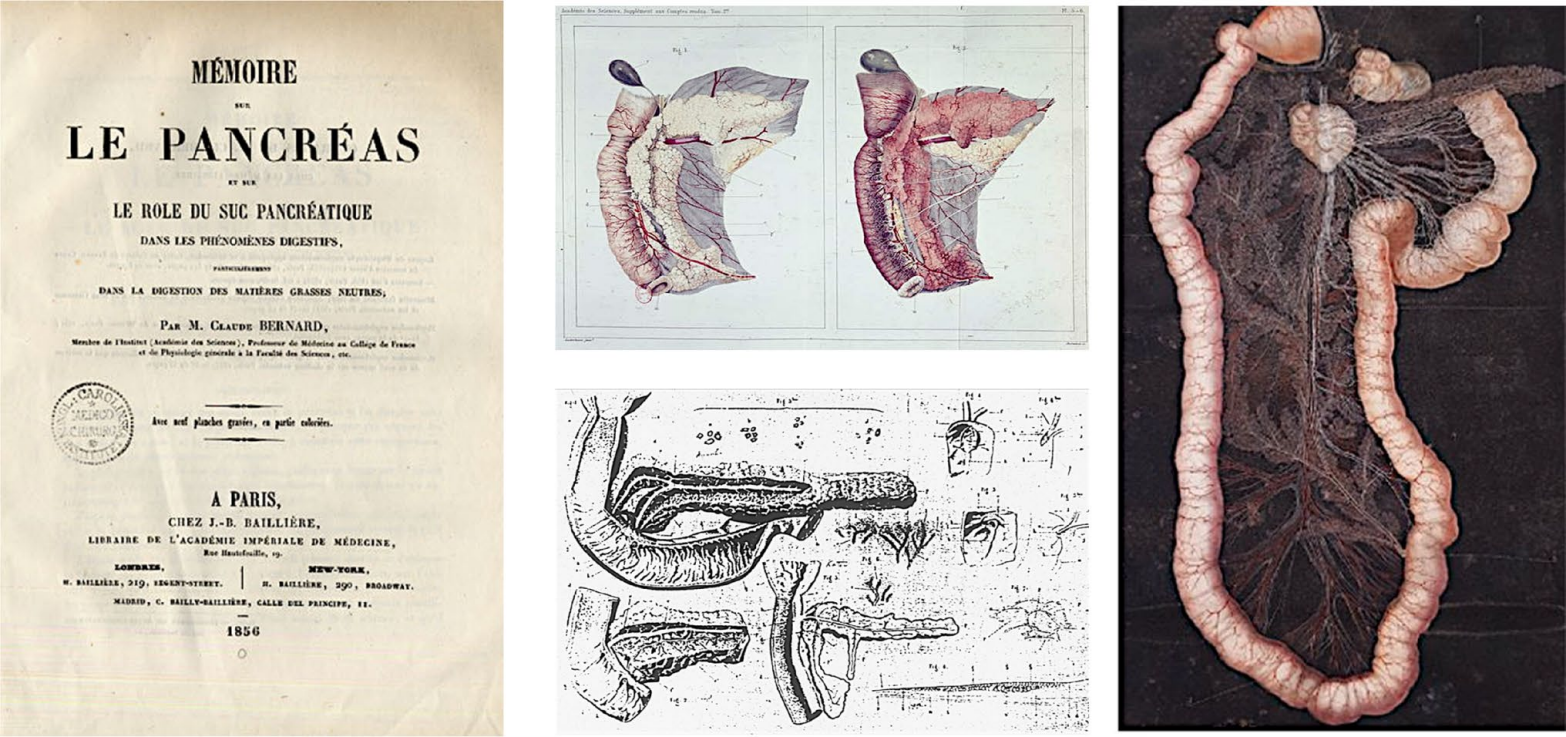
Bernard C (1856): “Mémoire sur le pancréas et sur le rôle du suc pancréatique dans les phénomènes digestifs”. First edition. Published in vol I of Supplement aux Comptes Rendus, pp 379–563. Selected plates [5]

In 1843 he defended his doctoral thesis, which was the first in a long series of publications on digestion and nutrition [4]. His complete work on the exocrine function of the pancreas, a classic document of pancreatic physiology that included experiments on pancreatectomised dogs, was published in 1856 (Fig. 2) [5].

## Claude Bernard (1813–1878): the internal environment (“milieu intérieur”) and the birth of endocrinology

Between 1846 and 1848, Bernard reported that sugar was present in the blood of normal animals, even in the fasting period. He also observed that the concentration of sugar in the liver was higher than in the portal vein. While sugar was found in very high amounts in the liver, it was practically absent in most organs [6, 7].

In March 1853 Bernard defended his second doctoral thesis, this time in the natural sciences. His experiments confirmed his idea that the liver was a glucose-synthesising laboratory [8]. He published an article on the isolation of hepatic glycogen in 1857, almost simultaneously with Christian Hensen, who demonstrated that glycogen was a carbohydrate [9].

Bernard hypothesised that, after intestinal absorption, sugar was converted to hepatic glycogen. He attributed excess secretion to diabetes, as a consequence of three possible processes: (a) hepatic failure of glycogen production; (b) exacerbated glycogenolysis; or (c) decreased sugar combustion, mainly in muscle [10]. Bernard refuted the hypothesis of the pancreatic origin of diabetes, observing that atrophy of the pancreas secondary to ligation of the pancreatic ductal system was not associated with experimental diabetes, [11–13]. Bernard's demonstration of the hepatic synthesis of glycogen and the hepatic release of glucose into the blood was the basis for his proposal of the "internal environment" (*milieu intérieur*) and homeostasis, anticipating the birth of endocrinology [14].

By detecting the presence of glucose in the cerebrospinal fluid, Bernard postulated that the excessive conversion of glycogen to glucose could be the consequence of brain lesions/alterations responsible for hyperglycaemia-glucosuria, similar to what was observed experimentally with the "piqûre diabétique". He stimulated the floor of the fourth ventricle with a needle, inducing a transient hyperglycaemia lasting less than 24 h. The connection between the vasomotor centre and a putative diabetic centre in the medulla would make possible an increased conversion of glycogen to glucose by virtue of a vasomotor disturbance in the liver, of neurogenic origin [15, 16].

As early as 1858, Bernard planned to write a treatise on experimental medicine. While convalescing from a dysenteric process in his native village of St Julien, near Lyon, he wrote a fundamental text in his scientific biography, *Introduction à l'étude de la médecine expérimentale*, creating for posterity the foundations of scientific research in medicine and the interaction between clinic and laboratory [17].

**Fig. 3:**
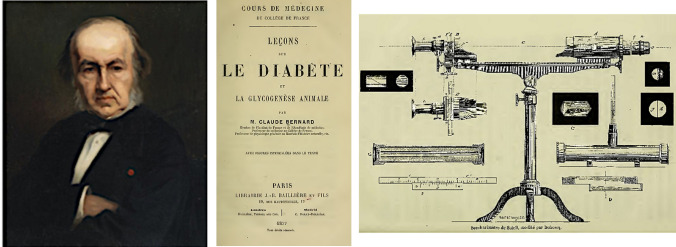
Claude Bernard. Oil painting by Charles Auguste Mengin (1870). (Internet Archives). Leçons sur le diabète et la glycogenèse animale. Sacarimeter used by Bernard (1877) [19]

Bernard was also interested in the pathogenesis of diabetic disease [12, 13, 18]. In 1877 he prepared 21 lectures on diabetes at the Collège de France (Fig. 3) [19].

## First description of the histology of the pancreas: Paul Langerhans (1847–1888)

Before 1869 the pancreas had been described as a racemose gland, containing infiltrated fat.

Berlin student Paul Langerhans, who was influenced by Rudolph Virchow and Julius Cohnheim, worked in Virchow’s laboratory at the Berlin Pathological Institute [20]. Langerhans’s early observations were made on rabbit, salamander, guinea pig, dog, cat, pigeon, snake, frog, hen and, finally, man [21]. In 1869, he reported his findings on the histology of human pancreas [22].

**Fig. 4:**
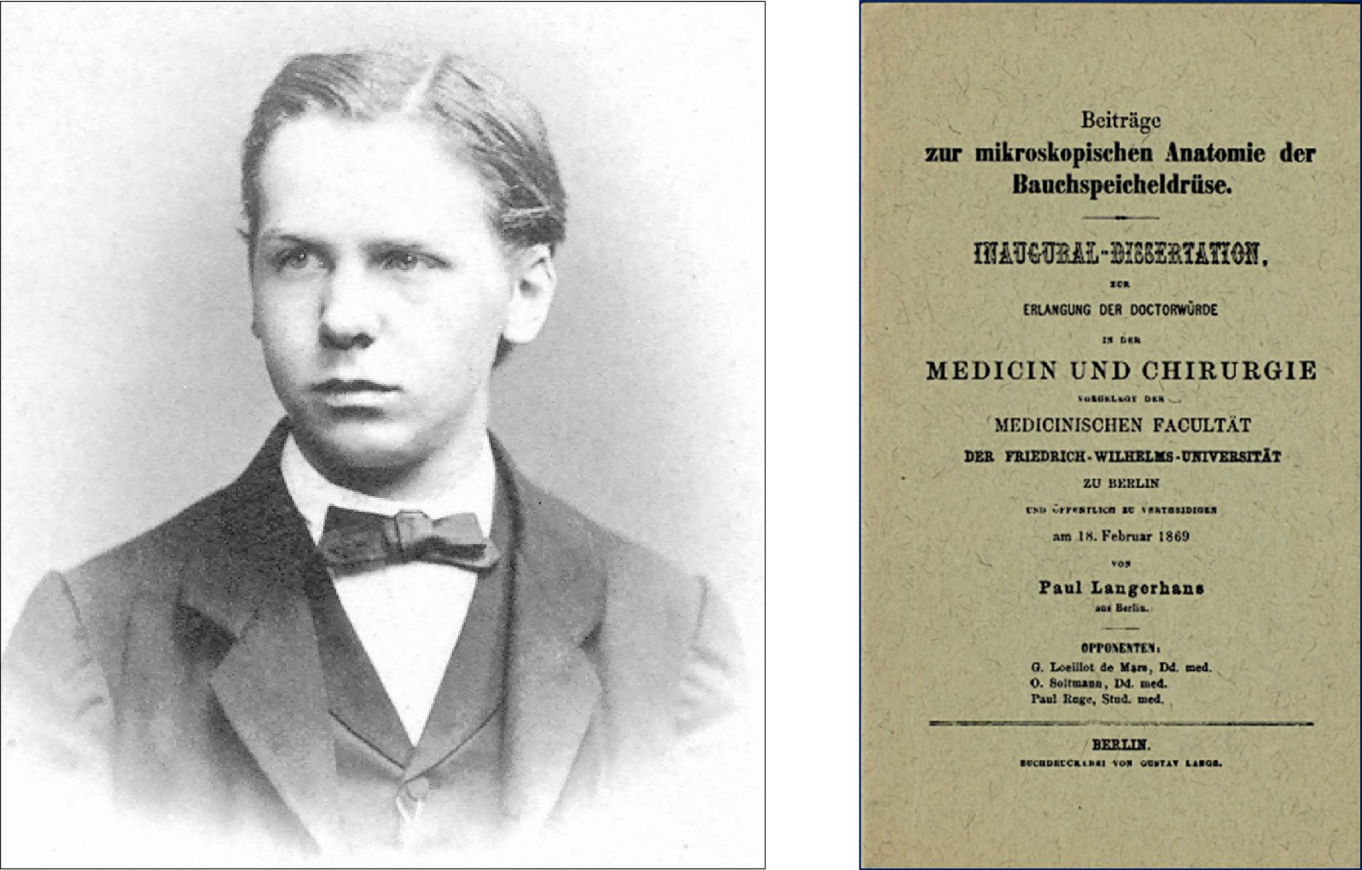
Photograph, Paul Langerhans (unknown author and date). Dissertation. University of Berlin, 1869 [23] [1]

In his doctoral thesis, Langerhans described in detail the ductal system of the gland by injecting Prussian blue dye and glycerine. In macerated fragments of the pancreas he discovered polyhedral cells with clear cytoplasm, grouped in small masses (Haüflein) of various sizes in diameter (0.1–0.25 mm), which, in contrast to the centroacinar cells, did not show any transition to the ductal epithelium. This investigation was the first comprehensive description of pancreatic histology (Fig. 4) [23].

Langerhans made it clear that he ignored the function of these cells [21–24]. The French histologist Edouard Laguesse immortalised Paul Langerhans 24 years later by giving his name to these cellular islets without an excretory duct [25].

Another area of interest for Langerhans was the skin. In 1868, he reported dendritic cells in the epidermis; the significance of these cells remained an enigma for over a century; it has been discovered that they are immunocompetent cells involved in many dermatological diseases [26].

In November 1871, Langerhans was appointed lecturer (*Privatdozent*) and two years later, Associate Professor of Pathology and Anatomy at the Faculty of Medicine, University of Freiburg. In September 1874 he was diagnosed with pulmonary tuberculosis. He resigned from his professorship and decided to spend the last years of his life in Madeira, where he died in 1888. [20].

## Diabetes mellitus: a pancreatic disease

The Swiss Johann Conrad Brunner (1653–1727) performed ligatures of the Wirsung duct, pancreatectomies and the combination of both procedures, publishing his findings in 1683. He was only able to perform incomplete pancreatectomies; it was impossible for him to resect the upper region of the gland due to its intestinal adhesion and profuse vascularisation, nor the neighbouring area of the duodenum. The dogs showed polyuria and polydipsia; they survived several months, with the exception of those that died of infectious processes. Brunner did not relate these findings to diabetes [1].

We owe the first allusion to the relationship between pancreas and diabetes to Richard Bright (1789–1858), who in 1833 reported a clinical case of pancreatic cancer with liver metastases associated with a syndrome of polyuria-polydipsia and concentrated urine with a "very sweet taste" [27].

**Fig. 5:**
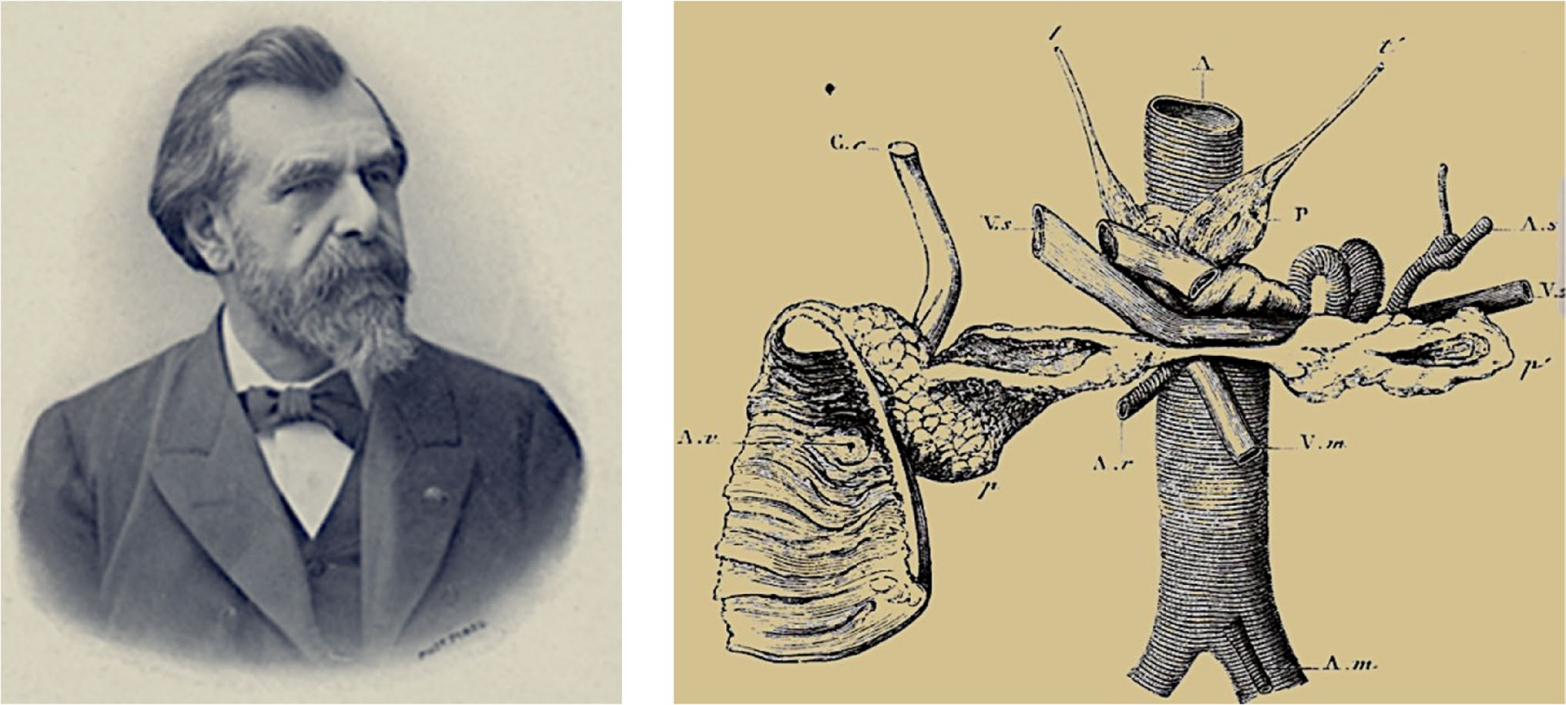
Étienne Lancereaux. Author and date unknown. The National Library of Medicine. Plate depicting atrophy of the pancreas in a case of severe diabetes [29]

**Étienne Lancereaux** (1829–1910) practised clinical medicine from 1869 in several hospitals in Paris as director of several Internal Medicine teams (Lourcone, 1874; St. Antoine, 1876; Pitié, 1878, and finally Hôtel Dieu, 1889). His main area of interest was the clinicopathological aspects of diabetes *mellitus*. A member of the French Academy of Medicine, he was elected president in 1903. In 1877 he coined the term *pancreatic diabetes* to describe two cases of young subjects who died with severe diabetes and showed pancreatic atrophy at autopsy (Fig. 5) [28].

Lancereaux made a clear distinction between *diabète maigre* and *diabète gras*. He considered diabetes as a syndrome rather than a disease [29–35].

Lancereaux contacted Bernard to discuss these findings in detail. At his insistence, the physiologist agreed to carry out further experiments and to administer pancreatic extract to pancreatectomised dogs. Unfortunately, Bernard died a year later without being able to fulfil his promise [1].

## Oskar Minkowski: the discovery of pancreatic diabetes (1889)

Oskar Minkowski (1858–1931) belonged to a family of Jewish descent from Eastern Europe, who made important contributions to the science of the time. Born in Alexoten, near Kaunas (now Lithuania), he studied medicine at Freiburg, Strasbourg and Könisberg, where he completed his doctorate in 1881. He went with his mentor, Professor Bernhard Naunyn, to the University of Strasbourg, where he received the appointment of Associate Professor in 1891. His academic career continued in Köln, Greisfald and finally in Breslau (Professor and Chair of the Department of Medicine), retiring in 1926. Among his many contributions in the field of diabetes *mellitus* were the identification of beta-hydroxybutyric acid in urine, the finding of decreased blood levels of carbon dioxide tension and the incorporation of alkalis in the treatment of diabetic ketoacidosis [36].

Minkowski combined great talent and intuition with surgical skills. “A lucky combination of serendipity, intelligence and surgical dexterity”, explained the legendary landmark of the development of diabetes after complete pancreatectomy in 1889 [37].

40 years after the discovery, Minkowski presented the analysis of the milestone experiment at the 25th Anniversary Meeting of the Medical Society of Rhein-Westphalen, on November 18, 1928, in Köln. Rachmiel Levine translated the conference from *Muenchener Medizinische Wochenschrift* (76:311–315; 22 February, 1929) and the text was reproduced by the American Diabetes Association in 1989 on the occasion of the 100th anniversary of the first publication by J. von Mering and O. Minkowski. We extracted here some paragraphs of this historical event [38]:

**Table:** 

(…) Had we realized that all previous attempts at pancreatectomy had led to nothing of note and that no less a person than Claude Bernard had stated that it was impossible for dogs to survive the total surgical removal of the pancreas, we would have certainly not dared to make a new attempt at this procedure. (…) We undertook our investigation for purposes other than to study the regulation of carbohydrate metabolism. (…) “I tried to tie all the ducts of the pancreas in order to show that neutral fat is absorbed more slowly than fatty acid, but I could not totally prevent the appearance of pancreatic juice in the lumen of the intestine”, said von Mering. (…) Why didn’t you extirpate the gland? I asked…Please, give me a dog and I will try to do a total extirpation of the pancreas…The same day von Mering transferred a dog to me…Von Mering and I removed the gland in toto; I endeavoured to do this under meticulously clean surgical conditions…We did not think of diabetes; hence, we did not test the urine for glucose. (…) On several occasions the dog emptied his bladder spontaneously on the floor and I reproached the lab assistant that the animal was not properly trained. “I did train him”, he stated, “but this animal is quite peculiar. No sooner does he empty his bladder completely when he has to urinate again and again”. I followed a subconscious hunch. I gathered a few drops of urine off the floor into a pipette and tested for glucose; it produced a strong reduction signifying more than 10% sugar content…	

**Fig. 6:**
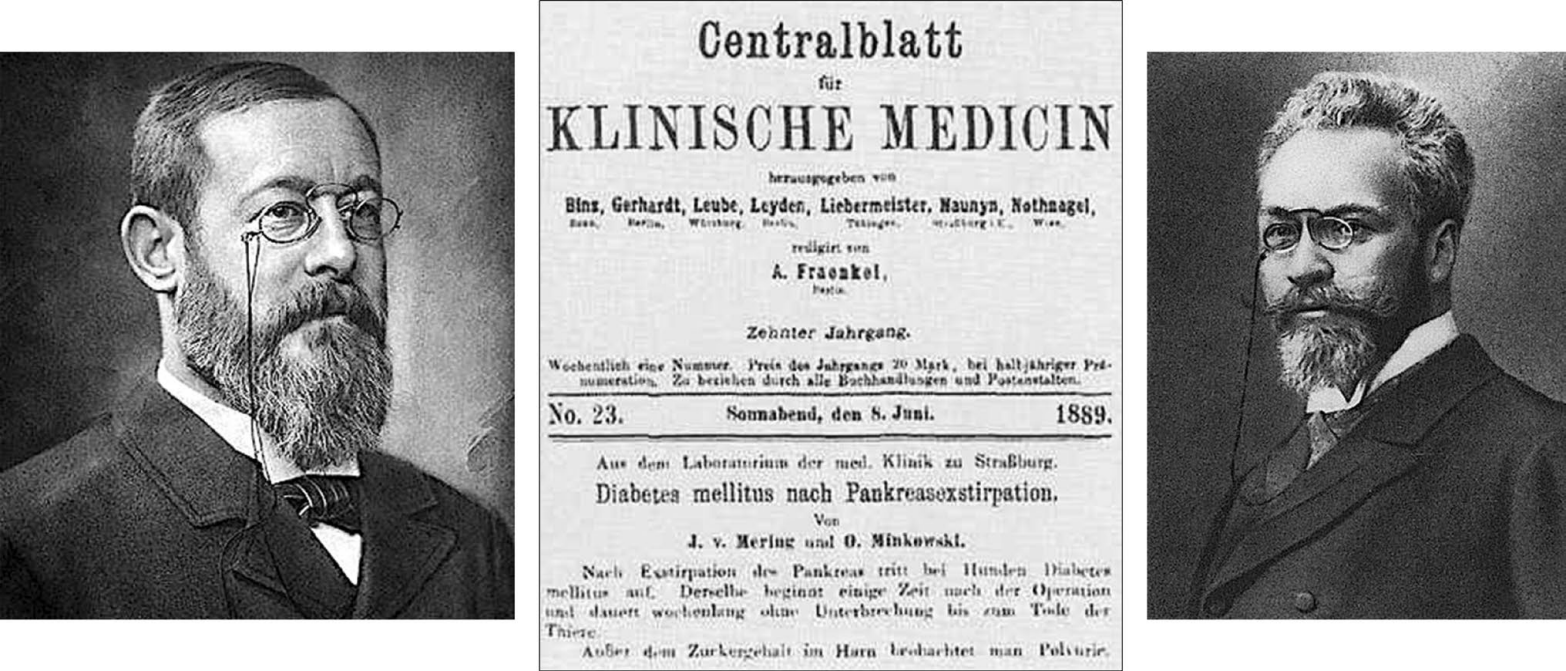
In 1889 Oskar Minkowski, with help from Joseph von Mering, discovered that diabetes is a pancreatic disease [39]

Minkowski confirmed the pancreatic origin of diabetes in experiments with three other dogs. The second and third animals died after two days due to duodenum necrosis; both developed glycosuria before they died. The fourth animal survived and from the second day after the operation showed a persistent diabetes. Minkowski presented the first paper at the Strasbourg Medical Society in May 1889, at the International Congress of Physiology in Basel and in a lecture at the Society of Naturalists in Heidelberg in September 1889. Von Mering and O. Minkowski published the first two reports under joint authorship [39, 40]. The first publication was a short paper in 1889 (Fig. 6) [39] and more extensive reports appeared in 1890, 1892 and 1893 [40–43]. The primacy of discovery undoubtedly belongs to Minkowski [44].

**Fig. 7:**
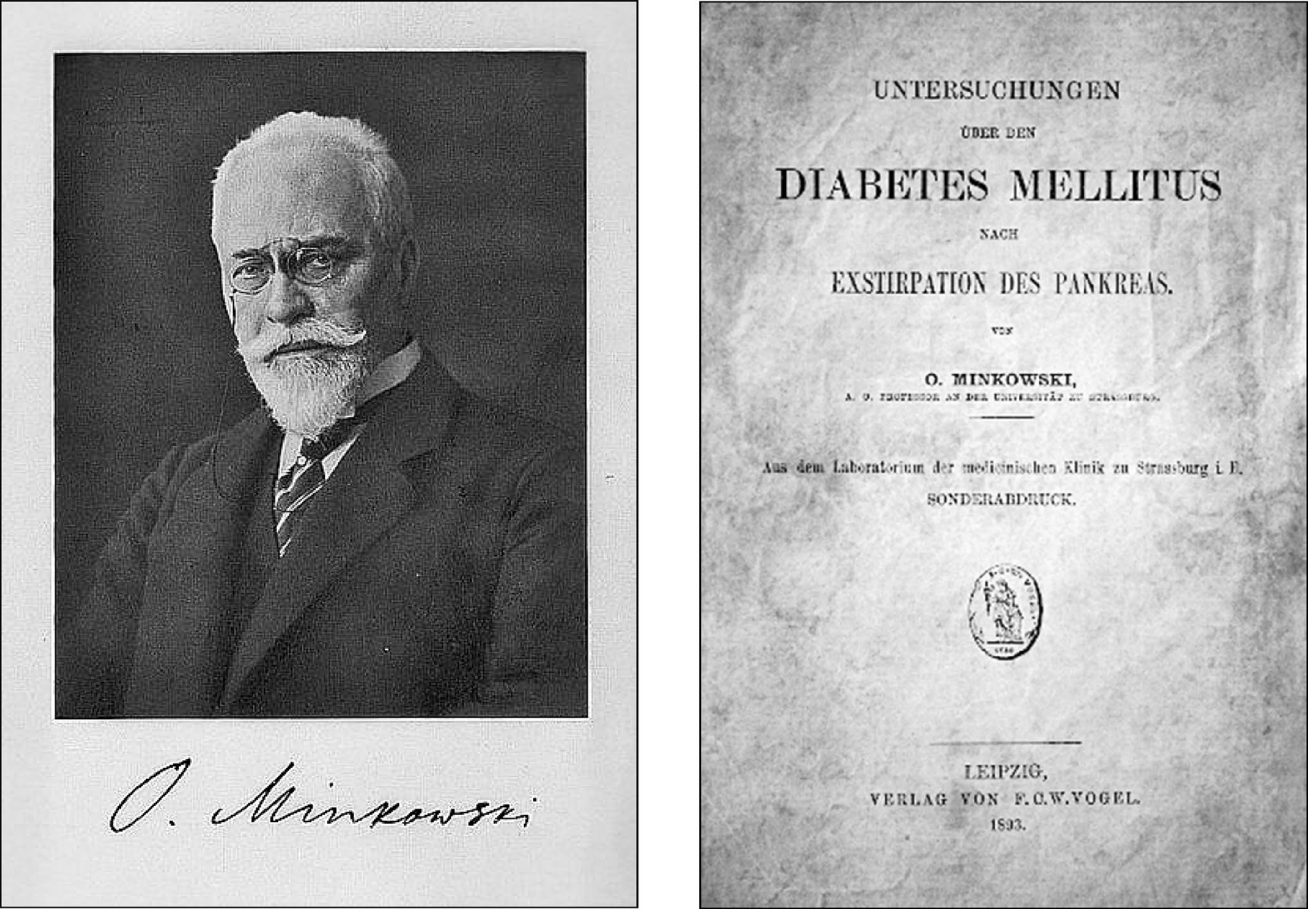
Left: Portrait of Oskar Minkowski. Naunyn-Schmiedebergs Archiv für Experimentelle Pathologie und Pharmakologie Collection BIU Santé-Licence ouverte. Unknown author. Right: Minkowski O (1893) Untersuchungen über den Diabetes mellitus nach Exstirpation des Pankreas. Leizpig: F.C.W. Vogel [42]

After the first experiment, von Mering did not participate in further research, directing his excellent academic career elsewhere. He was the discoverer of the drugs veronal and paracetamol and the glycosuric action of phloridzin. Oskar Minkowski provided the first conclusive evidence of the impact of the pancreas on diabetes (Fig. 7) and demonstrated that the disease is due to the *absence of the pancreatic active substance* that is transported by the bloodstream, a discovery considered the most important in the history of diabetes [37].

**Fig. 8:**
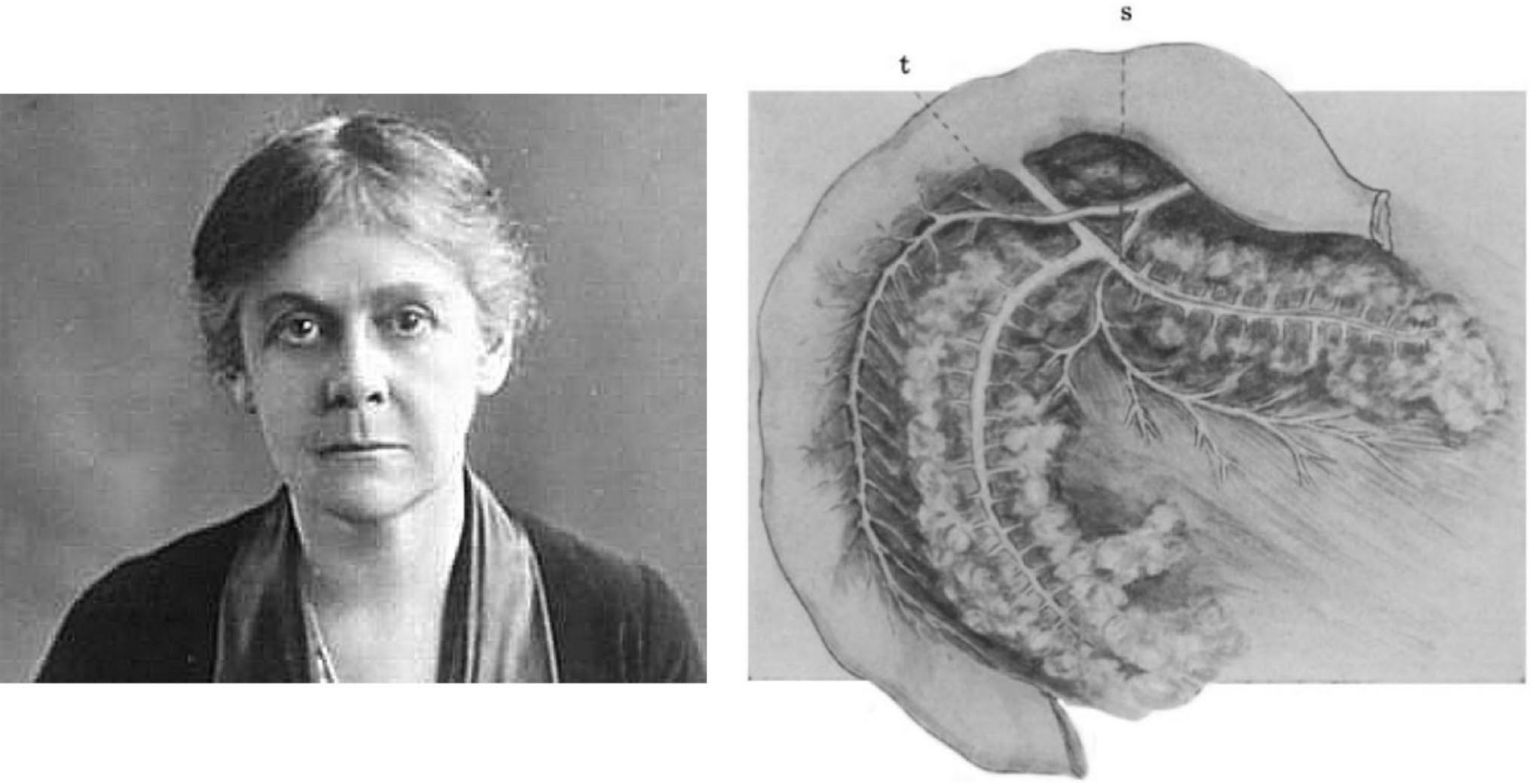
Picture of Lydia Maria Adams DeWitt. Unknown Author and Unknown Date. (Internet Archives). Dissection of the pancreas of the cat clearly reveals the ductal system and its relationship to the venous system (DeWitt, 1906) [46]

Between 1904 and 1906, at the Department of Medicine and Pathology of the University of Michigan (headed by George Dock), **Lydia Maria Adams DeWitt** (1859–1923) carried out interesting studies on the morphological and microscopic analysis of the endocrine and exocrine pancreas and on the pathophysiology of diabetes. She investigated the morphology and microscopic anatomy of acinar tissue and islets of Langerhans in various animal species (cats, rabbits, rats, guinea pigs, batrachians and birds) and in humans (Fig. 8) [45]. She obtained excellent glandular images using staining solutions to highlight the ductal system and vascularisation.

Animals survived up to 197 days after ligation of the ductal system. Acinar hypotrophy/atrophy ("degeneration") was not associated with glycosuria in any case in serial measurements over one month. As the disappearance of the acinar tissue and ductal system was greater than that of the connective tissue, the degenerated gland maintained its lobular appearance in most cases.

Dewitt also carried out histological and metabolic studies after the death of the animals. Dividing the pancreas into small fractions, she investigated the digestion of starch with iodine and Fehling's solution, the fat content by the emulsification process, and of protein content by Biuret's colorimetric test. She also determined the glycolytic activity of the pancreatic extract (Pavy method) before and after exposure to elevated temperatures for 24 h, using muscle tissue as a target. The results of these studies allowed her to conclude the following:

1. The histology of the islets of Langerhans showed no differences among the vertebrate species investigated.
2. The structure of the islets remained practically unchanged in the "degenerated pancreas".
3. The "pancreatic degeneration" was not accompanied by glycosuria in any case.
4. The islets of Langerhans release a secretion into the bloodstream that has no digestive function.
5. Secretion from the islets of Langerhans exerts a potent glycolytic effect, confirming its character as an "active principle" on carbohydrate metabolism [46].

**Eugene Lindsay Opie** (1873–1971) received his PhD in Medicine from Johns Hopkins Medical School. Under the tutelage of Wiliam H. Welch he remained at the institution as a fellow, assistant and instructor in pathology [47]. Quite soon, Opie won wide recognition for his outstanding observations related to disease of the pancreas. In 1899 he reported the relation between haemochromatosis and diabetes [48].

Opie described two forms of chronic inflammation of the gland: a) interlobular pancreatitis, comprising fibrous tissue proliferation, affecting the islets of Langerhans only in very advanced stage of the sclerotic process; this variety typically appears after the occlusion of the pancreatic duct; b) interacinar pancreatitis, showing more diffusely distributed fibrous tissue with affected islets as the remaining elements in association to atrophied cells [49].

**Fig. 9:**
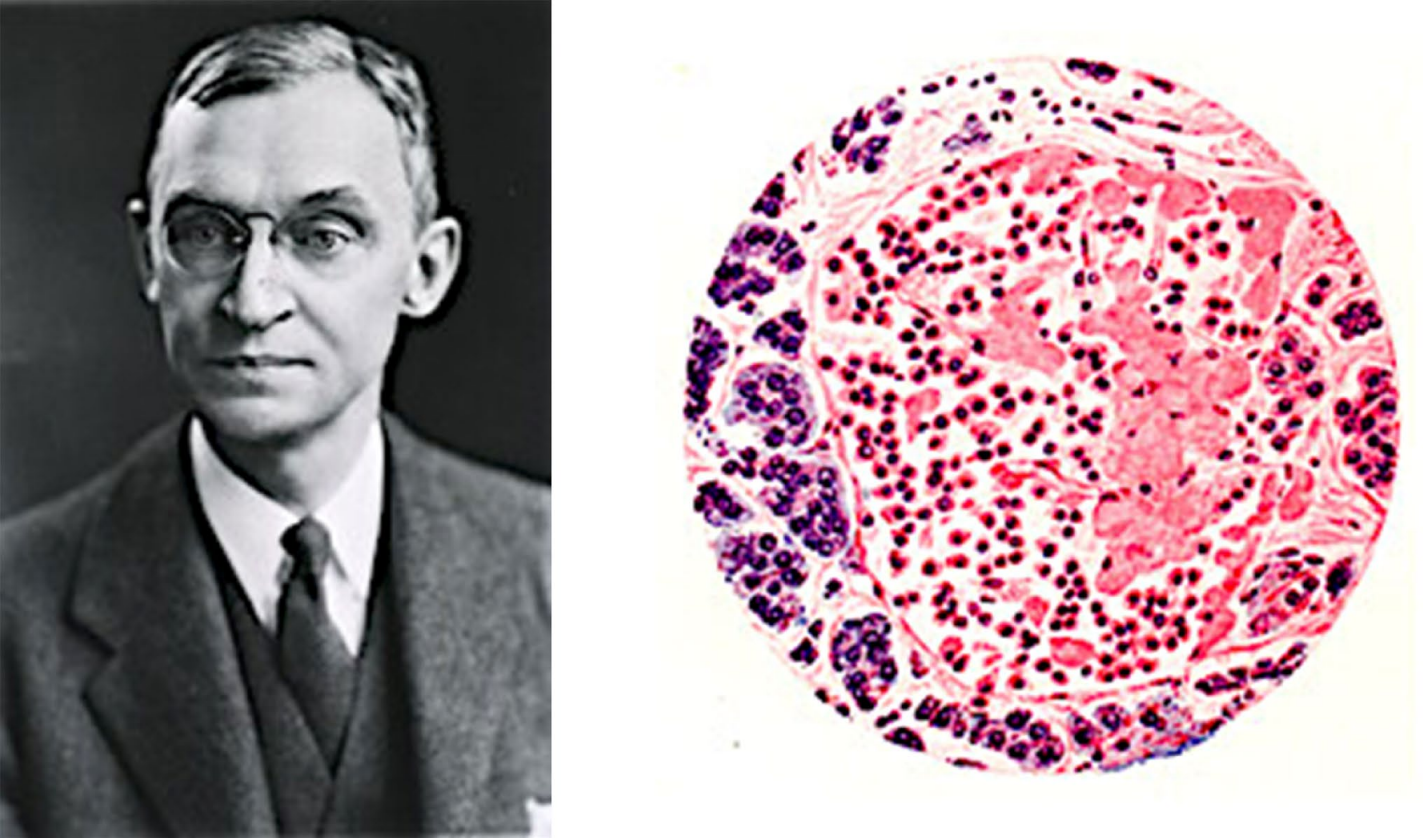
Portrait of Eugene L. Opie. Images from the History of Medicine (IHM). National Library of Medicine (NLM Image ID: B020236. http://resource.nlm.nih.gov/101424810 [1]. Drawing of the islands of Langerhans cells partly transformed into hyaline material (Opie, 1903) [52]

Opie was the first to describe hyaline degeneration of the islets of Langerhans, in which the parenchymatous cells are replaced by hyaline material [50]. This alteration is most evident in the tail of the gland. Hyaline transformation resides in the accumulation of a homogeneous material between the islet cells and the capillary wall that stains intensely with eosin and picric acid. Hyaline degeneration occurs as scattered globular masses. In some cases, the hyaline substance occupies almost the whole islet area. In certain cases of diabetes, Opie reported an infiltrative process unrelated to hyaline degeneration, indistinctly aggressive against acinar cells and islets, corresponding to foci of haemorrhagic necrosis, coinciding with an acute/subacute clinical course in a significant fraction of affected patients [51]. In 1903, at the age of 30, only five years after finished medical school, Opie published his classic book *Disease of the Pancreas* (Fig. 9) [52].

In 1904, Opie joined the Rockefeller Institute. Between 1910 and 1923 he became member of the faculty at Washington University Medical School in St. Louis, and in 1932, he became professor of Pathology at Cornell Medical School. In 1940 he returned to the Rockefeller Institute to concentrate on research, and he remained active into his nineties [1].

## Experimental diabetes: pioneering contributions of Emmanuel Charles Èdouard Hédon (1863–1933)

**Fig. 10:**
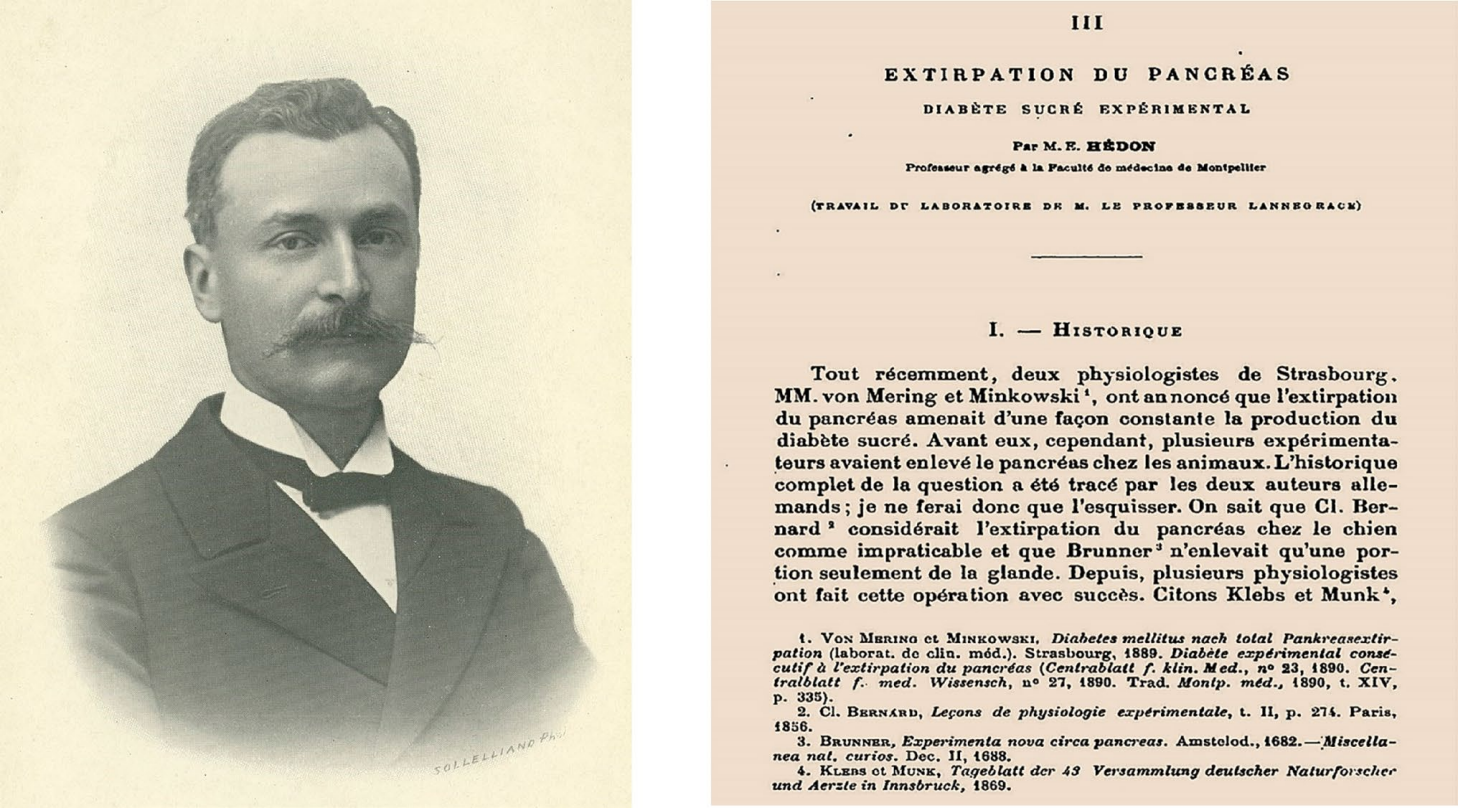
Emmanuel Hédon. Photograph of P. Sollelliano (Collection BIU Santé Medecine, Université de Paris). In 1891 he described his original technique of complete pancreatectomy of the dog, performed in two operative stages [53]

Emmanuel Hédon was born in Burie. He was professor of Physiology at the University of Montpellier and elected member of the French Academy of Medicine in 1914. In 1891, he described his two-stage pancreatectomy procedure, using the dog as an experimental animal (Fig. 10) [53].

Hédon confirmed Minkowski's criteria which considered the main operative rules in complete pancreatectomy to be: (a) ensure haemostasis; (b) preserve the vascularisation of the duodenum to prevent necrosis; (c) observe strict antisepsis. In all his operations he ligated the pancreaticoduodenal vascular trunk. In the first operative stage he made a central abdominal incision (above the linea alba) and exposed the duct of Wirsüng, close to the duodenal junction. After ligating the accessory duct, with the help of a cannula introduced into Wirsüng's canal, he injected molten paraffin [54].

Hédon then removed the entire vertical portion of the gland, which was easy to separate from the rest, and ligated its attachment to the head of the pancreas. After 8–15 days, he would again open the abdomen (incision at the level of the right hypochondrium) and perform the entire exeresis. The removed pancreas showed atrophy and sclerosis, with a loss of up to two-thirds of its original volume. Within 24–48 h of complete pancreatectomy, the animals showed glycosuria, increased azoturia, polyuria, polydipsia, and voracious appetite. Despite increased food intake, they lost weight and muscle mass. Operated animals survived for 15 to 30 days, occasionally up to 3 months. They did not die as a result of the surgical trauma but as a consequence of the disease caused by the removal of the pancreas. In all cases of complete ablation of the pancreas, severe glycosuria was detected within 24–48 h (average 50 g/L). After reaching the maximum, glycosuria decreased until death of the animal, disappearing two to three days earlier. Diabetes associated with complete pancreatectomy in the dog was similar to that known in man as "lean diabetes" [53].

In several lectures (9 April, 1 August and 4 October) in 1892, Hédon presented to the Société de Biologie (Paris) the details of the procedure and the results obtained with the subcutaneous autotransplantation of the pancreas, as well as the peculiarities of experimental diabetes.

The autotransplantation procedure consisted of fixing the lower portion of the pancreas under the skin of the dog's abdomen, preserving the vascular connection to ensure glandular nutrition, while adhesions between the pancreas and the subcutaneous cellular tissue were being formed. When the surgical wound healed, the pancreatic fragment only communicated with the abdominal cavity through two very thin vessels (artery and vein) that passed through the scar tissue of the wound. In the first days after the operation, the retention of pancreatic juice in the glandular channels caused the glandular fragment to increase in volume and form a tumour under the skin the size of an egg. Through the healed wound runs a small fistulous fragment through which pancreatic juice is poured. Over time the fistula closes and excretion is exhausted. If the dog with a subcutaneous pancreatic autotransplantation has the pancreatic remnant in the abdomen removed, glycosuria does not occur. If the graft is removed, severe glycosuria develops within hours and persists until death in a profound state of cachexia [55].

The liquid poured from the small fistula is analysed with the following protocol: (1) Addition of a few drops of the liquid to a paste of cooked starch and heating over the flame of a Bunsen burner; in a few seconds the saccharisation process takes place, and the addition of the Fehling solution shows its reducing capacity. (2) Stirring in a test tube a few drops of the liquid with a small volume of olive oil; a persistent emulsion is instantly generated and after several hours the mixture becomes acidic. (3) Introduction of small fragments of muscle tissue into the fistula fluid and heating of the mixture in an oven at 40 °C; after two hours digestion of the tissue takes place; the process is continued by adding half the volume of water and boiling it; the albumin is digested and the clean filtrate is precipitated by the addition of special reagents for peptones [56].

Hédon’s textbook *Physiologie normale et pathologique du pancréas* comprises his most relevant contributions to experimental diabetes, which expand on the descriptions of his research published in 1891–1892 [57].

Joseph H. Pratt praised Hédon’s contributions to experimental diabetes at the 64th Annual Meeting of the American Medical Association, held in 1910 in Saint Louis. In his words: “Hédon has asserted that the internal secretion of the pancreas will remain a hypothesis until it is possible to isolate from the gland a substance, the injection of which will check completely the diabetes of a depancreatized dog” [58].

Emmanuel Charles Èdouard Hédon was nominated four times for the Nobel Prize in Physiology or Medicine (three in 1926 and one in 1932) [59].

## Organotherapy of diabetes: initial stage

The discovery of internal secretions led to the origin of organotherapy or opotherapy, a procedure that uses extracts from animal organs to replace an absent or insufficient internal secretion in the organism.

In 1884, Victor Horsley demonstrated the induction of an experimental model of myxedema by removing the thyroid gland from an ape [60]. In 1891, George Murray administered a fresh extract of sheep thyroid hypodermically to a 46-year-old woman with florid myxedema [61]. The patient survived to the age of 74 years thanks to thyroid organotherapy, subsequently also successfully administered orally. Organotherapy of hypothyroidism with thyroid extracts represented one of the most important triumphs of hormone replacement therapy and scientific medicine.

Several research teams tested the administration of pancreatic extracts from various animal sources in experimental diabetes and in isolated cases in the clinic, mainly in Great Britain, using commercial pancreatic extracts administered orally or parenterally, with unsatisfactory results, published in 1893 [62–68]. In 1894, P. Watson Williams treated with pancreatic extracts a 15-year-old boy in the Bristol Royal Infirmary during approximately a month, without success. Then, pieces of sheep pancreas were subcutaneous implanted. The procedure triggered a diabetic coma and the boy died three days later. The case was reported as an overt failure (“we must guard against attributing a too important position to the pancreas as a factor in diabetes mellitus”) [69].

Leonidas Sobolev (1876–1919), a collaborator of L. P. Pavlov, observed that ligation of the pancreatic duct in the rabbit induced hyperplasia of the islets of Langerhans, a procedure that facilitated the anatomical isolation of the islets and the study of their properties without the presence of digestive ferments, facilitating the organotherapy of diabetes. To this end, he suggested the use of embryonic or early-aged islets, comparatively more developed than acinar tissue [70].

Between 1902 and 1904, John Rennie and Thomas Fraser, researchers at the University of Aberdeen, administered pancreatic extracts from bony fishes (teleosts) and cartilaginous skeleton fishes (elasmobranchs) for months to five patients with diabetes (clinically severe in four of them). In teleost fish, in particular, the islets of Langerhans are located independently of the exocrine pancreas. The islets were macerated in a mortar with a weakly acidic (acetic) solution at 40 °C and then filtered before daily oral administration of 4 g in three doses. The experience did not generate relevant results. One patient was additionally treated hypodermically with three daily doses of 1.5 g, also without significant results (the patient died in diabetic coma) [71].

## Marcel Eugène Émile Gley (1857–1930)

**Fig. 11:**
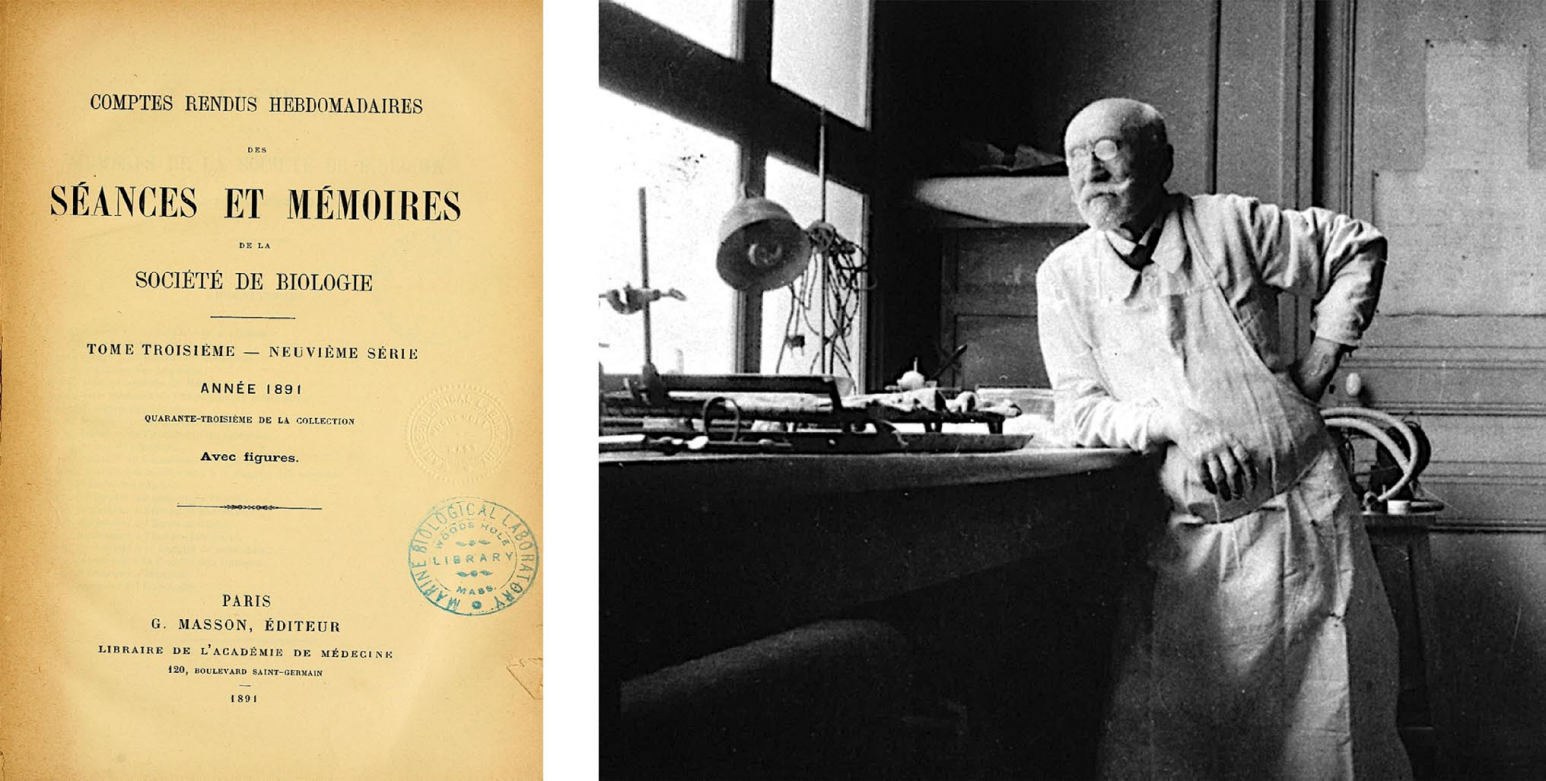
Eugène Gley. For many years he collaborated with the Société de Biologie (Paris) and its journal, C.R. Soc. Biol. Wellcome Collection Library, London, reference 127,961. Probable date: 1925

Eugène Gley was born in Espinal (France). He received his medical training at the universities of Montpellier and Nancy, where he defended his doctoral thesis in 1881. He entered the Collège de France, under the direction of Jules Marey, in 1880. Between 1886 and 1893 he was director of the clinical laboratory at the Hotêl Dieu. He obtained the post of *professeur agrégé de Phisiologie* at the Faculty of Medicine in Paris (Sorbonne) and in 1908 the chair of General Biology at the Collège de France. He collaborated for many years with the Société Française de Biologie (vice-president in 1897 and secretary in 1899), publishing numerous works in its official journal (Comptes Rendus des Séances et Mémoires de la Société de Biologie) (Fig. 11). A member of the French Academy of Medicine since 1903, he became its president in 1927. Shortly before his sudden death in Paris on 25 October 1930, he received the Osiris prize in Physiology from the Institut de France [72].

**Fig. 12:**
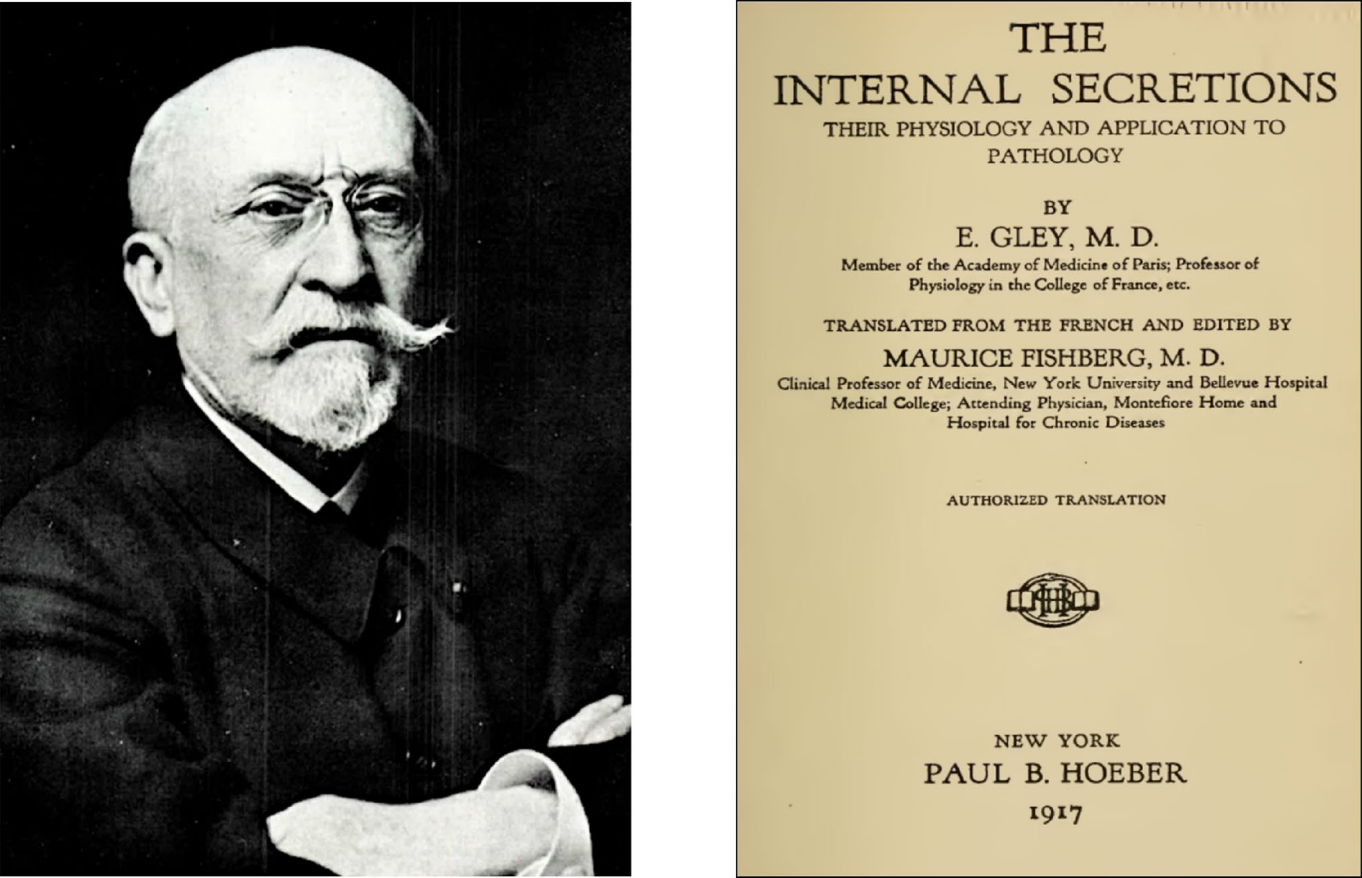
Gley’s treatise “The Internal Secretions (their Physiology and applications to Pathology)”, translated into English in 1917 by Maurice Fishberg, professor at New York University, has been described as being of historical interest. The photograph of the French professor comes from the Institute of Physiology of the University of Barcelona [1]. Probable date: 1919

In 1890, Gley demonstrated that thyroidectomised animals could survive if treated with aqueous extract of thyroid gland, anticipating its introduction into the clinic by G.B. Murray. He was also the first to understand and publish the physiological role of the parathyroid glands, as well as investigating the relationship between the adrenal glands and the autonomic nervous system. A worthy follower of Claude Bernard, his treatise on internal secretions is considered a most important document (Fig. 12).

Many researchers and historians consider Gley to be a cofounder of organotherapy and endocrinology [1]. His critical spirit regarding experimentation with organ extracts earned him antipathy among surgeons and clinical endocrinologists of his time who overindulged in organ transplantation or administration of glandular extracts with little scientific rigour [73].

Between 1881 and 1929 he published 524 papers [74]. He was particularly interested in physiology and organotherapy, mainly of the endocrine glands. Eugène Gley received a total of fifty-one nominations for the Nobel Prize in Physiology or Medicine (1921, 1925, 1926, 1928 and 1931), eleven of them in 1921 [75].

A group of selected works carried out by E. Gley qualifies him as undisputed authority on the induction of experimental diabetes and a pioneering scientist in therapeutic trials with pancreatic extracts in the pancreatectomised animal, which he initiated in 1890 with positive results. Within hours, pancreatectomised dogs developed glycosuria, ketonuria and azoturia, only in case of complete glandular ablation. Coincidental observations were confirmed in other animal species (pigs, cats, rabbits, frogs, turtles…). Shortly after surgery, the animals became voracious and showed polyuria and polydipsia. A dog weighing 7 kg excreted over 1000 ml of urine per day as opposed to 200–400 ml previously. In addition to glucose, the urine of animals with experimental diabetes contained high concentrations of acetoacetic acid, hydroxybutyric acid and acetone [76]. Several weeks later, the dogs developed serious malnutrition in association to muscle weakness, important weight and hair loss, and died of marasmus [77]. Gley found that the regulatory function of the pancreatic antidiabetic principle extended to the liver. Pancreatic glycosuria did not occur if the animal was hepatectomised at the same operative time (experiments with frogs). J. Thirolox and É. Gley performed subcutaneous autotransplantations in 12 previously pancreatectomised dogs, following the procedure described by Hédon. The surviving animals developed transient glycosuria (4–5 days) and the "tumour" generated by the autotransplantation increased considerably in size. Graft exeresis led to the rapid onset of marked hyperglycaemia and within a few weeks the animals died with diabetic cachexia, as reported by Hédon.

**Fig. 13:**
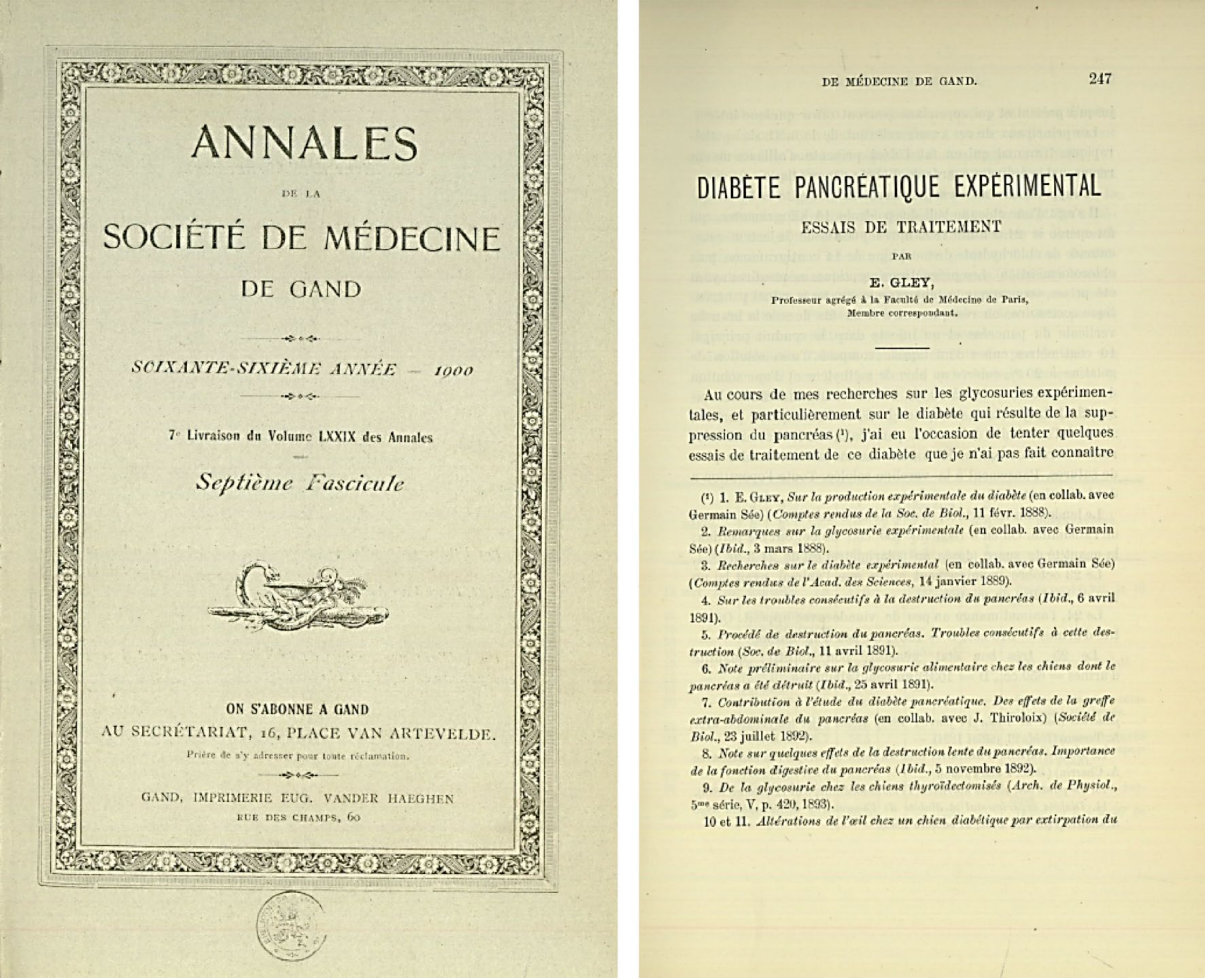
E. Gley published in 1900 series of experiments performed in his laboratory since 1892 describing in detail the metabolic actions of the pancreatic extract [“Diabète Pancreatique Expérimental: Essais de Traitement”] [79]

These experiments convinced Gley, definitively, that diabetes was indeed a pancreatic disease and not a liver disease, as Claude Bernard thought. To demonstrate the existence of the active principle of the islets of Langerhans, capable of preventing experimental diabetes, Gley reproduced the previous experiments of Minkowski and Bernard. To eliminate the exocrine function of the gland, he ligated the accessory pancreatic duct and injected into Wirsüng's canal mixtures of olive oil and glycerine, or sodium carbonate and glycerine, or gelatine and melted tallow at 40 °C. To check that the product penetrated the entire gland, he used gelatine coloured blue or tallow coloured violet. He waited for the time required for the 'degeneration' of the acinar tissue to take place and prepared aqueous extracts of the 'sclerosed pancreas. On December 16, 1892, Gley administered the aqueous extract of "degenerated pancreas" intraperitoneally to a dog pancreatectomised in two stages (Hédon's procedure), which reduced the daily absolute glycosuria from 115.5 to 38.6 g, an experiment he described in detail in 1900 (Fig. 13) [78].

Statement and sealed letter from E. Gley to the Société de Biologie de France.

**Fig. 14:**
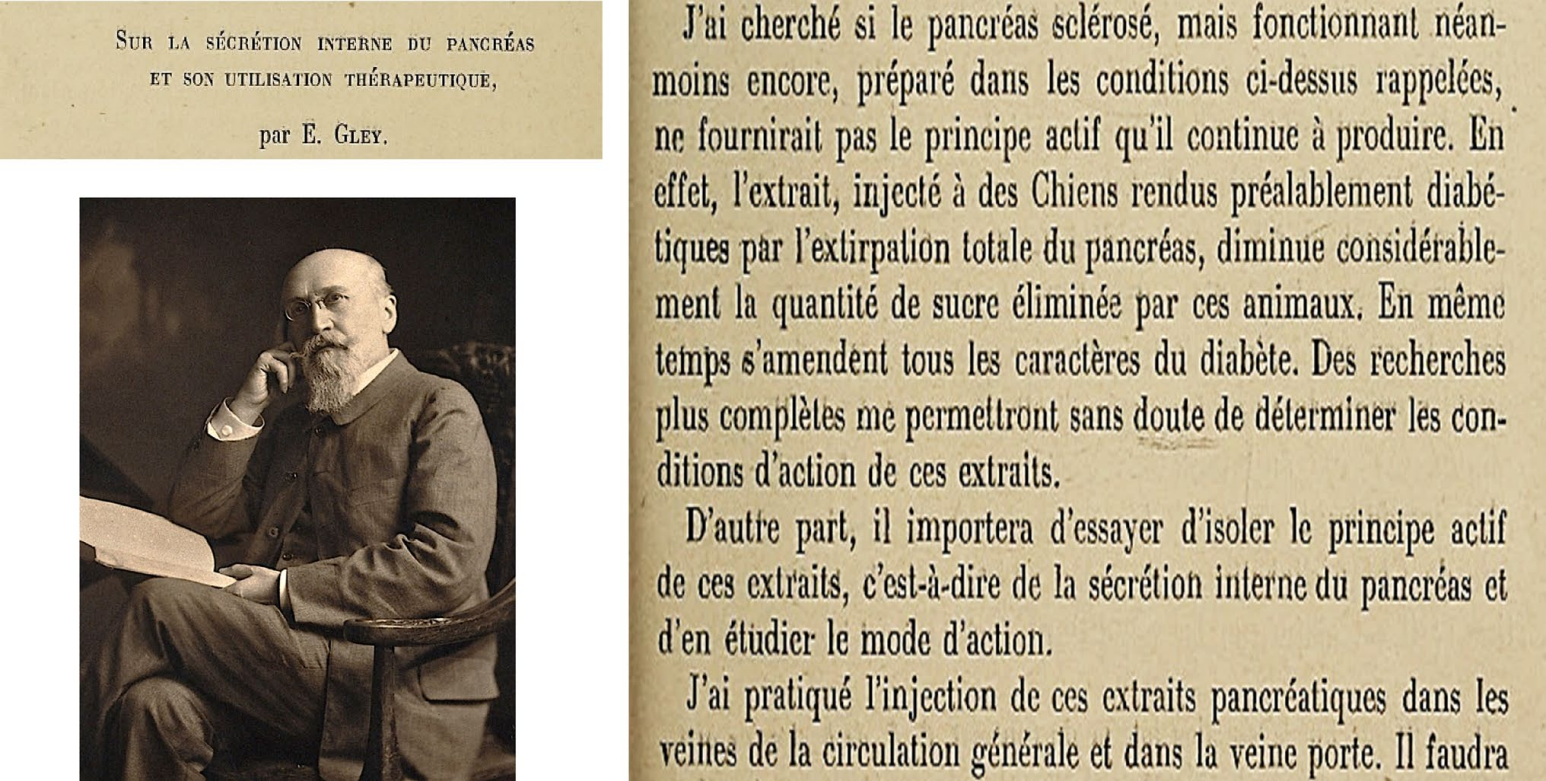
E. Gley: Detail, enlarged, of the text of the sealed letter "Sur la sécrétion interne du pancréas et son utilisation thérapeutique" (1905). Gley's photograph, date unknown, is from the Wellcome Collection Library (date unknown; author, Lafayette Ltd., public domain)

When the first experiences of the University of Toronto research team led by J.J.R. Macleod were published, claiming that extracts from pancreatic glands ("degenerated", foetal and adult) reduced hyperglycaemia and glycosuria in dogs with post-pancreatectomy diabetes, as well as glycaemia in healthy rabbits, the French physiologist, on 23 December 1922, staged one of the most eccentric acts in the history of science before the Assembly of the Société de Biologie. Gley asked to make public a document he had submitted to the Society's secretariat on 20 February 1905. The short paper was entitled: “Sur la sécrétion interne du pancréas et son utilisation thérapeutique”. In this paper, Gley summarised the experiments he started in 1890 with "extracts of degenerated pancreas", obtained by occluding the excretory ducts of the gland. He added that parenteral injections of these extracts reduced urinary excretion in pancreatectomised dogs, improving their clinical condition. In his letter, Gley claimed that the "sclerosed pancreas" provides the active principle, since the extract injected into diabetic dogs by total removal of the pancreas significantly decreased the amount of sugar eliminated. At the same time, the features of diabetes were corrected. "I've been injecting these pancreatic extracts into the veins of the peripheral circulation and the portal vein. It will be important to try to isolate the active principle of these extracts from the internal secretion of the pancreas, and to study its mode of action". Gley concluded the paper by stating that these experiments had been conceived at least twelve years earlier and had to be interrupted in 1900–1901 because of other commitments. However, in the doctoral thesis defended by J. Lafon, in 1906, directed by Gley, both supported the theory that the internal secretion of the pancreas is essential for hepatic glycogen synthesis and in experiments conducted in 1909 and 1910, Gley reproduced similar investigations by administering intraperitoneal injections of the extract; he observed in pancreatectomised dogs a reduction of urinary glucose from 14.0 to 2.3 g/L (Fig. 14) [80].

Although in the documentation so extravagantly delivered by Gley in 1905 the protocol is not sufficiently detailed (a frequent circumstance in those years), it is plausible to admit that his final results (considerable decrease in glycosuria and substantial improvement of diabetic symptoms) were correct [81].

J.J.R. Macleod gave credit to Gley's research. In his book *Carbohydrate metabolism and insulin*, published in 1926, he wrote the following about Gley's scientific activities in relation to the antidiabetic hormone of the pancreas [82]:

**Table:** 

“At the meeting of the Société de Biologie, held in Paris on 23rd December, 1922, to commemorate the centenary of the birth of Pasteur, Professor E. Gley requested that an envelope, deposited by him in February, 1905, be opened and read the inside document entitled “Sur la sécrétion interne du pancréas et son utilisation thérapeutique”. In this communication, after referring to his earlier researches in which it was shown that the destruction of the pancreas in situ does not lead to diabetes, Gley states that it is probable, as indicated by the words of Laguesse, that this was because the islets of Langerhans remained intact. (…) He therefore prepared extracts from sclerosed remains of pancreas, and found them to diminish considerably the sugar in the urine of depancreatised dogs, and to alleviate all the other diabetic symptoms. Gley then indicate his intentions to isolate the active antidiabetic principle, to study its mode of action and to see whether the extracts could be used on man, either subcutaneously or by mouth. Because of other researches, these problems were laid aside. Previous to depositing this sealed package, Gley had contributed valuable observations concerning the effects on depancreatised dogs of extracts of the entire pancreas prepared in various ways.”	

## The role of the priority rule in science

Robert K. Merton, founding father of the Sociology of Sciences, defined the *Priority Rule* as the credit given to the individual or group of individuals who first made the discovery, documented by an *original publication* or filing the *successful application for a patent* [83, 84].

Already in the seventeenth century, the Royal Society of London, and later on, the Academy of Sciences of Paris and other scientific societies accepted, as an alternative to the Priority Rule, a signed and dated document showing an original description, sealed in an envelope and deposited in the secretary, to be read in a future meeting of the institution [1, 85].

This was the case of Eugène Gley with his sealed letter deposited by him in the Secretary of the Society of Biology in Paris on February 20, 1905. In this communication Gley stated that since 1891 he had carried out multiples experiments comprising: a) induction of diabetes after complete pancreatectomy, b) development of pancreatic extracts from sclerosed remains of the gland, and c) administration of these extracts to pancreatectomised dogs. The extracts were able to reduce the amount of sugar excreted and alleviated diabetic symptoms.

## European research, the cradle of the discovery of the antidiabetic hormone

In 1889, Oskar Minkowski discovered the development of diabetes after complete pancreatectomy. Eugène Gley confirmed this landmark discovery in 1891. One year later (1892), Gley demonstrated that the atrophy of the acinar pancreas did not induce experimental diabetes in the dog. Furthermore, after multiple experiments carried out during the following eight years, Marcel Eugène Èmile Gley, a worthy follower of Claude Bernard, cofounder of organotherapy and endocrinology, published in 1900 the metabolic effects of the pancreatic extract developed in his laboratory: reduction of urinary excretion of glucose, acetoacetic acid, hydroxybutyric acid and acetone. These experiments directly demonstrated, for the first time in history, the existence of the antidiabetic hormone of the pancreas.

## References

[1] de Leiva-Hidalgo A (2022) Organotherapy of diabetes mellitus (1889–1923). Controversies on the priority about the discovery of the antidiabetic hormone. PhD Dissertation, Faculty of Medicine, University of Valencia

[2] Howard JM, Hess W (2002). History of the pancreas: Mistery of a Hidden Organ.

[3] Barona JL (1989) Bernard. Antología. Barcelona, Ediciones Penísula

[4] Bernard C (1843) Du suc gastrique et son rôle dans la nutrition. PhD Dissertation, Paris, Rignoux. https://archive.org/details/dusucgastriqueet00bern/mode/2up?ref=ol&view=theater

[5] Bernard C (1856). Mémoire sur le pancréas et sur le rôle du suc pancréatique dans les phenomènes digestifs, particulièrement dans la digestion des matières grasses neutres.

[6] Bernard C (1848). De 1’origine du sucredans 1’économie animale. Arch Gén Méd.

[7] Bernard C (1850). Sur une nouvelle fonction du foie chez l’homme et les animaux. C R hébd Acad Sci.

[8] Bernard C (1853) Recherches sur une nouvelle fonction du foie considéré comme organe producteur de matière sucré chez l’homme et les animaux. Thèse presentée à la Faculté des Sciences de Paris, L. Martinet. https://www.biusante.parisdescartes.fr/histmed/medica/cote?extulyonun6030

[9] Hensen CAV (1857) Ueber die Zuckerbildung in der Leber Verh phys med Gesellsch Würzburg 7: 219. https://archive.org/details/101750353.nlm.nih.gov/mode/2up

[10] Bernard C (1867). Nouvelles recherches éxperimentales sur les phenómenes glycogéniques du foie. Mém Soc Biol.

[11] Bernard C (1849). Chien rendus diabétiques. C R Soc Biol.

[12] Bernard C (1849). Destruction du pancréas pendant la vie chez le chien. C R hébd Soc Biol.

[13] Bernard C (1854). Sur la destruction des glandes au moyen d’injections de matières grasses. C R hébd Soc Biol.

[14] Bernard C (1879). Leçons sur les Phénoménes de la Vie Communs aux Animaux et aux Végétaux.

[15] Bernard C (1858) Leçons sur la physiologie du système nerveux. Course de Médecine du Collège de France. Paris, Baillière et fils. https://gallica.bnf.fr/ark:/12148/bpt6k77329n

[16] Binet L (1949). Centenaire d’une découverte de Claude Bernard: diabète sucré par piqûre nerveuse. La revue des Deux Mondes.

[17] Bernard C (1861). Introduction à l’étude de la médecine expérimentale.

[18] Bernard C (1859) Leçons sur sur les propriétés physiologiques et les altérations pathologiques des liquids de l’organisme. Paris: Baillière. https://archive.org/details/leonssurlespro02bern

[19] Bernard C (1877) Leçons sur le diabète et la glycogenèse animal (Troisième Leçon). Paris: Baillière. https://archive.org/details/leonssurlediab00bern/mode/2up

[20] Hausen BM (1988) Die Inseln des Paul Langerhans. Eine Biographic in Bildern und Dokumenten, Wien, Berlin: Ueberreuter-Wiss-Verl

[21] Barach JH (1952). Paul Langerhans 1847–1888. Diabetes.

[22] Morrison H (1937) Paul Langerhans: contributions to the microscopic anatomy of the pancreas (Berlin 1869) with an english translation and an introductory assay. Bull Hist Med 5 (January 1937) Baltimore: John Hopkins University Press

[23] Langerhans P (1869) Beiträge zur Mikroskopischen Anatomie der Bauchspeicheldrüse. Dissertation, Medizinische Fakultät der Fiedrich-Wilhelms-Universität zu Berlin. https://insulin.library.utoronto.ca/islandora/object/insulin%3AT10076

[24] Campbell WR (1958). Men and books: Paul Langerhans, 1847–1888. Can Med Ass J.

[25] Laguesse E (1893). Sur la formation des îlots de Langerhans dans le pancréas. C R Soc Biol.

[26] Langerhans P (1868) Ueber die Nerven der menschlichen Haut. Archiv für pathologische Anatomie und Physiologie und für klinische Medicin 44(2):325–327. 10.1007/BF01959006

[27] Bright R (1833). Cases and observations connected with disease of the pancreas and duodenum. Med Chir Transact.

[28] Lancereaux E (1877) Notes et réflexions à propos de deux cas de diabète sucré avec altération du pancréas. Bull Acad Méd 2e série 6:1215

[29] Lancereaux E (1880). Le diabète maigre: ses symptômes, son évolution, son pronostic et son traitement; ses rapports avec les altérations du pancréas. Etude comparative du diabète maigre et du diabète gras. Coup d'oeil rétrospectif sur les diabetes. L'Union médicale.

[30] Lancereaux E (1891) Les diabètiques; les gras et les maigres. Diabète constitutionnel; diabète pancréatique. Gazelle Médicale 409

[31] Lancereaux E (1892) Sur la pathogenie du diabète pancréatique (en collaboration avec le Dr. Thiroloix). Académie des Sciences, Semaine Médicale 324

[32] Wright JR Jr, McIntyre L (2020) Misread and mistaken. Etienne Lancereaux’s enduring legacy in the classification of diabetes mellitus. J Med Biog 10.1177/0967772020914797 10.1177/0967772020914797 32279606

[33] Lancereaux E (1897) Pathologie générale du pancréas. J de Médecine Interne 71

[34] Lancereaux E (1899) Étude générale des affections du pancréas. Dans: Traité des Maladies du Foie et du Pancréas. Paris 781–795

[35] Lancereaux E (1899) Solénites pancréatiques; lithiase pancréatique. Dans: Traité des Maladies du Foie et du Pancréas. Paris 978–1010

[36] Minkowski O (1884). Ueber das vorkommen von oxybuttersäure im harn bei diabetes mellitus. Arch. Exp. Path. Pharm.

[37] Luft R (1989). Oskar Minkowski: Discovery of the pancreatic origin of diabetes, 1889. Diabetologia.

[38] Minkowski O, Levine R (1989). Historical development of the Theory of Pancreatic Diabetes. Diabetes.

[39] Von Mering J, Minkowski O (1889). Diabetes mellitus nach Pankreasextirpation. Zentralbl Klin Med.

[40] Von Mering J, Minkowski O (1890). Diabetes mellitus nach Pankreasextirpation. Arch Exp Path Pharm.

[41] Minkowski O (1892). Weitere Mittheilungen über den Diabetes mellitus nach Exstirpation des Pankreas. Berl Klin Woechenschr.

[42] Minkowski O (1893) Untersuchungen über den diabetes mellitus nach Exstirpation des Pankreas. Leizpig: F.C.W. Vogel

[43] Minkowski O (1893). Untersuchungen über den diabetes mellitus nach exstirpation des pankreas. Arch Exp Path Pharm.

[44] Houssay BA (1952). The discovery of pancreatic diabetes. Role Oskar Minkowski Diabetes.

[45] DeWitt LMA (1904–1905) Preliminary report of experimental work and observations on the areas of Langerhans in certain mammals. Am J Anat 4:8.

[46] DeWitt LMA (1906). Morphology and physiology of areas of Langerhans in some vertebrates. J Exp Med.

[47] Kidd JG (1971). Eugene Lindsay Opie, MD, 1873–1971. Am J Pathol.

[48] Opie EU (1899). A case of haemochromatosis. The relation of haemochromatosis to bronzed diabetes. J Exp Med.

[49] Opie EU (1901). On the relation of chronic interstitial pancreatitis to the islands of Langerhans and to diabetes mellitus. J Exp Med.

[50] Opie EL (1901). The relation of diabetes mellitus to lesions of the pancreas. Hyaline degeneration of islands of Langerhans. J Exp Med.

[51] Opie EL (1901). The etiology of acute hemorrhagic pancreatitis. Bull Johns Hopkins Hosp.

[52] Opie EL (1903). Disease of the pancreas. Its cause and nature.

[53] Hédon E (1891). Extirpation du pancréas. Diabète sucré experimental Arch Méd Exp.

[54] Hédon E (1891). Sur les phénomènes consécutifs à l’altération du páncreas déterminée par une injection de paraffine dans le canal de Wirsüng (avec note presentée par E. Gley). C R Soc Biol.

[55] Hédon E (1892). Greffe sous-cutanée du pancréas: ses resultats au pint de vue de la theorie du diabète pancréatique. C R Soc Biol.

[56] Hédon E (1892). Fistule pancréatique. C R Soc Biol.

[57] Hédon E (1901). Physiologie normale et pathologique du pancréas.

[58] Pratt JH (1910). The relation of the pancreas to diabetes. JAMA.

[59] Nobel Foundation (2015) Nomination database. Emmanuel Hédon. Nobel Media AB. https://old.nobelprize.org/nomination/archive/show_people.php?id=3985

[60] Horsley VAH (1884). A recent specimen of artificial mixedema in a monkey. Lancet.

[61] Murray GR (1891). Note on the treatment of myxedema by hypodermic injections of an extract of the thyroid gland of a sheep. Br Med J.

[62] Battistini F (1893). Due casi di diabete mellito curati con inezioni di estratto pancreatico. Gior Accad di med di Torino.

[63] Mackenzie HWG (1893). The treatment of diabetes mellitus by means of pancreatic juice. Br Med J.

[64] Wood N (1893) The treatment of diabetes by pancreatic extracts. Br Med J 1:64 10.1136/bmj.1.1672.64 PMC2402433 20754001

[65] Wills WA (1893). The treatment of diabetes by pancreatic extracts. Br Med J.

[66] White WH (1893) On the treatment of diabetes mellitus by feeding on raw pancreas and by the subcutaneous injection of liquor pancreaticus. Br Med J i:452–452 10.1136/bmj.1.1679.452 PMC2402761 20754086

[67] Sibley WK (1893). On the treatment of diabetes mellitus by feeding on raw pancreas. Br Med J.

[68] Marshall AL (1893). Treatment of diabetes by pancreatic extracts. Br Med J.

[69] Williams PW (1894). Notes on diabetes treated with extracts and by grafts of sheep’s pancreas. Br Med J.

[70] Sobolev LV (1902). Die Bedeutung der Langerhansschem Inseln. Arch Path Anat.

[71] Rennie J, Fraser T (1907). The islets of Langerhans in relation to diabetes. Biochem J.

[72] Medvei VC (1993). History of clinical endocrinology: a comprehensive account of endocrinology from earliest times to present day.

[73] The Lancet (1930) The late Prof. Gley. Lancet 216(5593). 10.1016/S0140-6736(01)09805-1

[74] E.A.S.S. (1933). Eugène Gley (1857–1930). Proc R Soc Edinb.

[75] Nobel Foundation (2015) Nomination Database. Eugène Gley. Nobel Media AB. https://www.nobelprize.org/nomination/archive/show_people.php?id=3484

[76] Gley É (1891). Procédé de destruction du pancréas. Troubles consécutifs a cette destruction. C R Soc Biol.

[77] Gley É (1892). (1892) Note préliminaire sur quelques effets de la destruction lente du pancréas: importance de la fonction digestive du pancréas. CR Soc Biol.

[78] Gley E, Thiroloix J (1892). Contribution à l’etude du diabète pancréatique. Des effets de la greffe extra-abdominale du pancréas. C R Soc Biol.

[79] Gley E (1900). Diabète pancréatique expérimental. Essais de traitement. Ann Soc Méd Gand.

[80] Gley E (1922). Action des extraits de pancréas sclerosé sur des chiens diabétiques (par extirpation du pancréas). C R Soc Biol.

[81] Pestel M (1972) Le cinquanteneire de la découverte de l’insuline: E. Gley, précurseur de F.G. Banting et C. H. Best. Nouv P Méd, 1(22):1527–1528 4556521

[82] Macleod JJR (1926). Carbohydrate metabolism and insulin.

[83] Merton RK (1957). Priorities in scientific discoveries: a chapter in the sociology of science. Am Sociol Rev.

[84] Merton RK (1973). The sociology of science: theoretical and empirical investigations.

[85] Strevens M (2003). The role of the priority rule in science. J Phil.

